# Synthesis of Oligodeoxynucleotides Using Fully Protected Deoxynucleoside 3′-Phosphoramidite Building Blocks and Base Recognition of Oligodeoxynucleotides Incorporating *N*^3^-Cyano-Ethylthymine

**DOI:** 10.3390/molecules15117509

**Published:** 2010-10-27

**Authors:** Hirosuke Tsunoda, Tomomi Kudo, Akihiro Ohkubo, Kohji Seio, Mitsuo Sekine

**Affiliations:** Department of Life Science, Tokyo Institute of Technology, 4259 Nagatsuta-cho, Midori-ku, Yokohama, Japan; E-Mails: htsunoda@bio.titech.ac.jp (H.T.); tkudo@bio.titech.ac.jp (T.K.); aohkubo@bio.titech.ac.jp (A.O.); kseio@bio.titech.ac.jp (K.S.)

**Keywords:** protecting group, oligodeoxynucleotide synthesis, cyanoethylation

## Abstract

Oligodeoxynucleotide (ODN) synthesis, which avoids the formation of side products, is of great importance to biochemistry-based technology development. One side reaction of ODN synthesis is the cyanoethylation of the nucleobases. We suppressed this reaction by synthesizing ODNs using fully protected deoxynucleoside 3′-phosphoramidite building blocks, where the remaining reactive nucleobase residues were completely protected with acyl-, diacyl-, and acyl-oxyethylene-type groups. The detailed analysis of cyanoethylation at the nucleobase site showed that *N*^3^-protection of the thymine base efficiently suppressed the Michael addition of acrylonitrile. An ODN incorporating N3-cyanoethylthymine was synthesized using the phosphoramidite method, and primer extension reactions involving this ODN template were examined. As a result, the modified thymine produced has been proven to serve as a chain terminator.

## 1. Introduction

Much attention has been focused recently on chemically synthesized oligodeoxyribonucleotides (ODNs) for use in biochemical and therapeutic studies [1,2,3]. Many modified ODNs have, therefore, been synthesized to improve their original sequence specificity, stability, hybridization ability, enzyme resistance, and various functional properties. Demand for effective syntheses of longer ODNs has also increased since the total synthesis of a bacterial gene of *Mycoplasma genitalium* was achieved by Gibson *et al.* [4,5], who utilized extensive stepwise ligations of medium-size ODNs. At present, the chemical synthesis of ODNs seems limited to a one hundred nucleotide-length level. This limitation is due to the fact that side reactions of the synthesis increase as the ODN chain length increases. Among the most serious side reactions observed is the cyanoethylation of the base residues during the deprotection step. Several improved procedures to avoid this side reaction have been reported. From a different viewpoint, however, this side reaction could be avoided altogether through the full protection of all reactive functional groups on the base moieties. To date, no such example of a full protection strategy with the phosphoramidite method has been reported, although as reported by our group, such strategies were employed in the phosphotriester approach. In this paper, we report the synthesis of ODNs using the full-protection mode and discuss the problems associated with this strategy.

The phosphoramidite approach for the chemical synthesis of ODNs is an established procedure [6,7,8,9] that has achieved high reliability in coupling efficiency, practicality, and versatility. Through the use of appropriately protected monomer building blocks, side reactions on the nucleobases have been minimized. The 2-cyanoethyl group has been commonly utilized for the protection of internucleotidic phosphate groups because of the ease of deprotection with the use of concentrated NH_3_ [10,11,12]. However, this protecting group generates the toxic and potentially carcinogenic compound acrylonitrile through *β*-elimination in the deprotection step [13,14]. The generated acrylonitrile has also been proven to be reactive towards the amino or imido groups on the nucleobases [14,15,16,17,18] to give addition products. It has also been reported that the imido NH group of the thymine residue is predominantly cyanoethylated over the other nucleobases (A, G, and C) [19]. In fact, Ravikumar has reported that ODN derivatives, cyanoethylated at the thymine residue, were isolated as side products when concentrated NH_3 _(aq) was used for the deprotection step [20]. Some researchers have recently studied the synthesis of ODNs without cyanoethylation at the nucleobase site because oligonucleotide products contaminated with cyanoethylated species would affect the chemical and pharmaceutical properties when used as DNA probes and nucleic acid drugs, respectively. Beaucage *et al*. has used the 4-[*N*-(2,2,2-trifluoroacetyl)amino]butyl group in place of the 2-cyanoethyl group for protection of the phosphate group [21]. The 4-[*N*-(2,2,2-trifluoroacetyl)amino]butyl group has no addition ability after the deprotection step because the protecting group is converted to a cycloaminoalkane derivative through the deprotection process. Wada *et al.* reported that the formation of *N*^3^-cyanoethylthymidine was efficiently suppressed by using nitromethane as a scavenger under the deprotection conditions [22]. Chang *et al.* indicated that the use of *t-*butylamine was efficient for avoidance of alkylation by acrylonitrile at the deprotection step [23].

**Figure 1:**
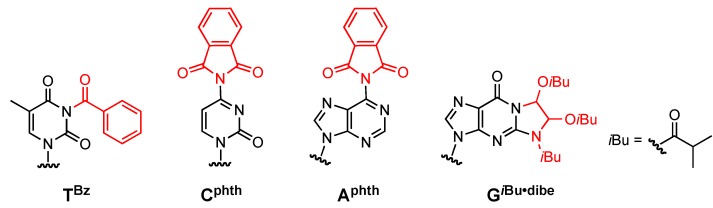
Fully protected monomers.

In this study, we synthesized ODNs with the focus of preventing the cyanoethylation at the nucleobase site through the use of fully protected monomers. The three kinds of deoxynucleoside 3′-phosphoramidite building blocks (C, A, G) were completely protected by acyl type protecting groups at the amino group of the nucleobases, as shown in Figure 1. In the thymine residue, the benzoyl (Bz) group was used to block the addition reaction. In the cytosine residue, the phthaloyl (phth) group was used to completely mask the two protons of the 4-amino substituent, because it was known that 4-*N*-benzoylcytidine is only slightly reactive toward acrylonitrile [21]. On the other hand, the adenine and guanine residues were comparatively non-reactive towards cyanoethylation in comparison to the thymine residue. The adenine and guanine moieties, however, were protected with phthaloyl and isobutyryloxyethylene (*i*Bu·dibe) groups, respectively, to entirely eliminate the possibility of the cyanoethylation side reaction.

In this paper, we report the synthesis of ODNs using these fully protected monomers. In addition, the stabilities of the fully protected base derivatives under various conditions were also examined with the focus of whether these protecting groups could be effective in avoiding the acrylonitrile-mediated Michael addition reaction. Lastly, we report the base recognition ability of *N*^3^-cyanoethylated T, when introduced into DNA, based on *T*_m_ experiments and DNA polymerase-mediated insertion reactions.

## 2. Results and Discussion

### 2.1. Synthesis of Fully Protected Deoxynucleoside 3′-Phosphoramidite Building Blocks

**Scheme 1:**
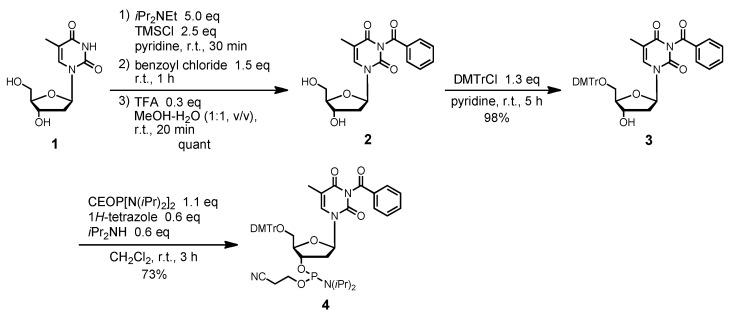
Synthesis of fully protected 2′-deoxythymidine derivative **4**.

To evaluate the deactivation of the fully protected nucleobases towards cyanoethylation with acrylonitrile, we synthesized fully protected phosphoramidite units, as previously reported [24,25,26,27,28,29,30,31,32,33,34]. The benzoyl group was selected as a full protecting group for the thymine residue [24,25]. The *N*^3^-protection of 2′-deoxythymidine by reaction with benzoyl chloride gave compound **2** quantitatively via the transient *O*-trimethylsilylation. The tritylation of compound **2** with DMTrCl, as per standard procedure, produced the 5′-protected product **3** in 98% yield. The phosphitylation of **3** with bis(diisopropylamino)(2-cyanoethoxy)phosphine produced the fully protected phosphoramidite unit **4** in 73% yield (Scheme 1).

**Scheme 2:**
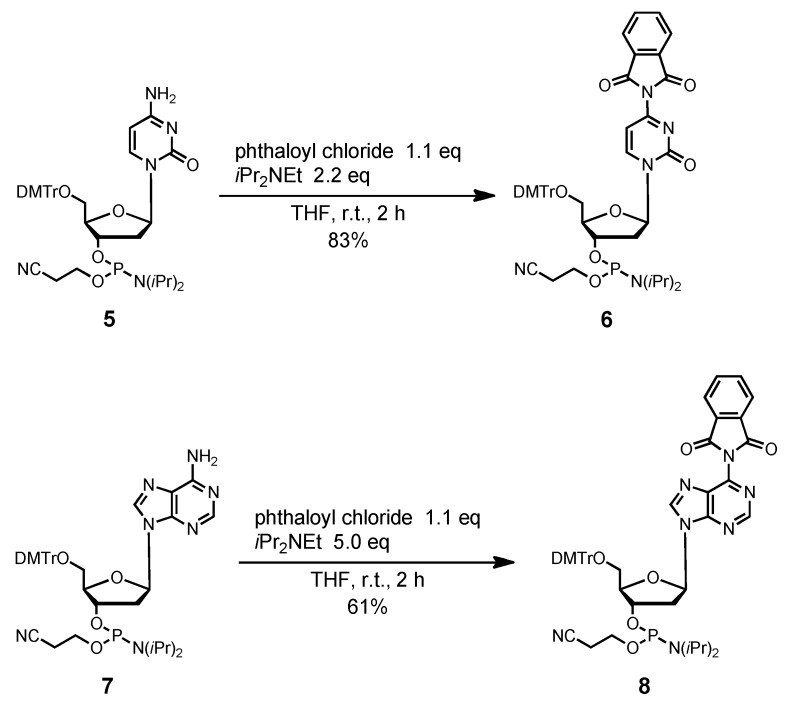
Synthesis of fully protected 2′-deoxycytidine derivative **6** and 2′-deoxyadenosine derivative **8**.

For the full protection of the cytosine and adenine residues, the phthaloyl group was utilized [26,27,28,29,30]. This protecting group enabled us to protect the two protons of the amino group of the C or A base simultaneously via formation of a five-membered ring. It was found that 2′-deoxyadenosine derivatives protected with the phthaloyl group exhibited significantly greater suppression of depurination under acid conditions than when protected with the simple benzoyl group. The phthaloyl group also possesses a unique property that, even when ring opening occurred, re-cyclization could be performed during the capping reaction with acetic anhydride. In this study, we developed a new route to the amidite units using selective deacylation of the commercially available 4-benzoyldeoxycytidine 3′-phosphoramidite derivative, followed by acylation of the resulting N-free species **5** with phthaloyl chloride [31]. Thus, the desired phosphoramidite building block **6** was obtained in 83% yield (Scheme 2). In a similar manner, the 6-*N*-phthaloyldeoxyadenosine 3′-phosphoramidite derivative **8** was synthesized in 61% yield by treatment of the N-free derivative **7** with phthaloyl chloride in the presence of diisopropylethylamine in THF.

**Scheme 3:**
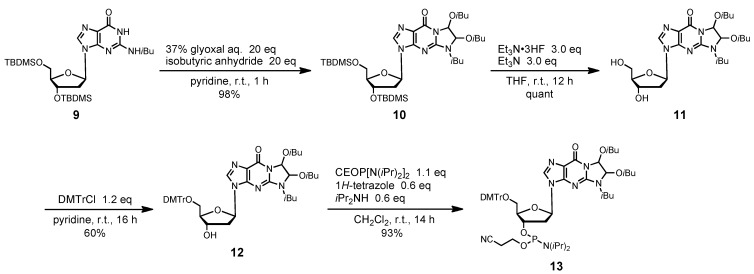
Synthesis of fully protected 2′-deoxyguanosine derivative **13**.

For full protection of the guanine base, we used a 1,2-bis(isobutyryloxy)ethylene (*i*Bu·dibe) group as an additional protecting group with the widely used isobutyryl group on the 2-amino substituent. This double protecting mode was used for the synthesis of G-rich oligodeoxynucleotides in the phosphotriester approach, and proved to effectively avoid the side reactions associated with the guanine base [32,33,34]. Moreover, the *i*Bu·dibe group could be simultaneously removed under the deprotection conditions of the other *N*-acyl protecting groups at T, C, and A. In this study, we synthesized the phosphoramidite unit of deoxyguanosine, protected with the *i*Bu·dibe group (Scheme 3). 3′,5′-Bis(t-butyldimethylsilyl)-2-isobutyryl-2′-deoxyguanine derivative **9**, having an isobutyryl group at the 2-*N*-amino group, was allowed to react with excess glyoxal and isobutyric anhydride in pyridine, which formed the fully protected derivative **10** in 98% yield. When the *N*-unprotected 2′-deoxyguanosine derivative was used as the starting material in this reaction, the desired compound **10** could not be obtained, but a complex mixture containing various glyoxal adducts was formed. Treatment of compound **10** with Et_3_N·3HF in THF gave the desilylated product **11**. Tritylation of **11** with DMTrCl, as per usual conditions, produced the 5′-protected product **12** in 60% yield. The phosphitylation of **12** with bis(diisopropylamino)(2-cyanoethoxy)phosphine produced the fully protected phosphoramidite unit **13** in 93% yield.

### 2.2. Evaluation of the Stability of the Fully Protected Nucleobases toward Cyanoethylation

We introduced the fully protected phosphoramidite units of T^Bz^, dC^phth^, dA^phth^, and dG*^i^*^Bu·dibe^ into a resin linked to UnyLinker™ in order to evaluate the resistance of the fully protected base moieties to the addition reaction of acrylonitrile under the conditions required for removal of the cyanoethyl group in the solid phase. Subsequently, each monomer loaded resin was treated with 100 equiv of acrylonitrile in DBU/CH_3_CN, which corresponded to the conditions estimated when a 100mer ODN was synthesized. To compare the stability of the fully protected monomers, stability of the common protected monomers [T, 4-*N*-acetyl-dC (dC^ac^), 6-*N*-phenoxyacetyl-dA (dA^pac^), and 2-*N*-(4-isopropyl)phenoxyacetyl-dG (dG*^i^*^Prpac^)] was also examined under the same conditions.

**Figure 2:**
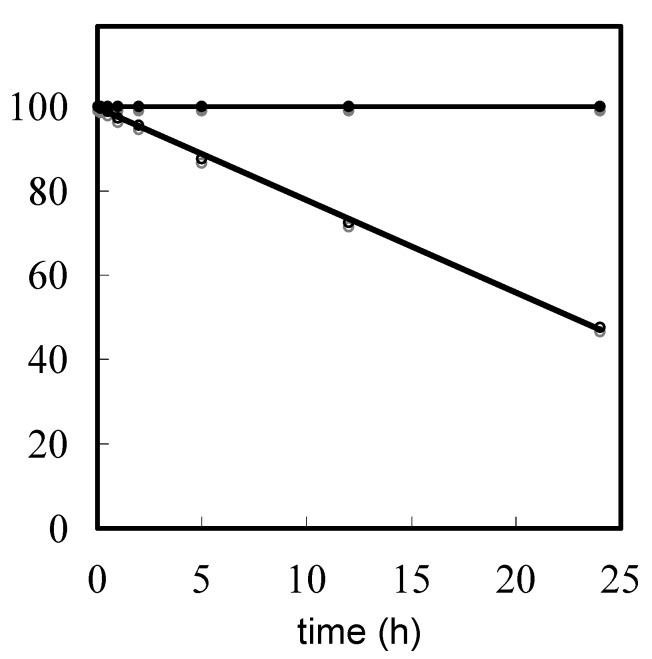
The remaining amount (%) of T or T^Bz^ in its reaction with acrylonitrile. Open and filled circles refer to the remaining amounts (%) of T and T^Bz^ in use of T and T^Bz^, respectively. Conditions: 100 equiv of acrylonitrile in DBU/CH_3_CN.

Cyanoethylation for common protected T, which is the most reactive compared to the other bases, was observed after 1 min by HPLC analysis. The *N*^3^-cyanoethylated derivative (T^CE^) of thymidine increased as time passed and was formed in 52% yield after 24 h. The time course of the remaining T monomer in the reaction of T or T^Bz^ with acrylonitrile is shown in Figure 2. Results indicated that T decreased linearly in a time-dependent manner. In contrast, when T^Bz^ was used in this evaluation, T^CE^ was not detected by HPLC analysis within the 24 h period. Therefore, protection of T at the *N*^3^-amino group proved to be very effective for suppression of cyanoethylation of T.

**Figure 3:**
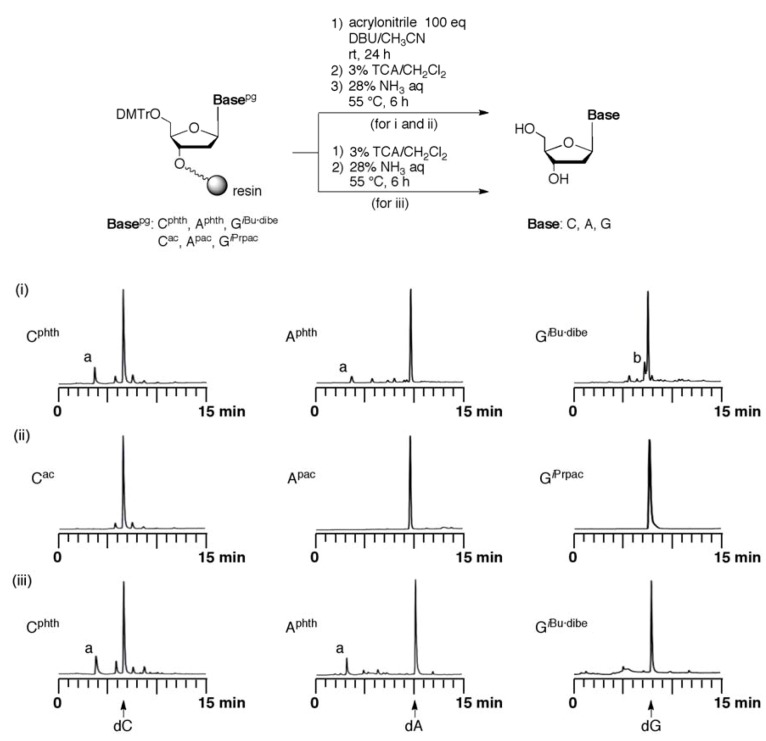
Analysis of cyanoethylation of (i) the fully protected monomers (C^phth^, A^phth^, and G*^i^*^Bu·dibe^) and (ii) the common protected monomers (C^ac^, A^pac^, and G*^i^*^Prpac^) by reverse-phase HPLC; (iii) Analysis of deprotection of the fully protected monomers by concentrated NH_3_ at 55 °C for 6 h.

Cyanoethylation of the other fully protected monomers (C^phth^, A^phth^, and G*^i^*^Bu·dibe^) did not occur within 24 h under the same conditions as those used for the ODN synthesis using the common protected monomers (Figure 3i). In the case of C^phth^ and A^phth^, the minor product (peak a) was phthalamide resulted from the ammonolysis of the phthaloyl group. In the case of G*^i^*^Bu·dibe^, the side product (peak b) was observed ahead of the peak of dG. This product is not an alkylated product but an incompletely deprotected G residue because the product (peak b) was decreased by prolonged treatment with concentrated NH_3_. To examine the stability of G*^i^*^Bu·dibe^ in DBU/CH_3_CN in detail, we carried out the reaction of the G*^i^*^Bu·dibe^ derivative **10** with DBU/CH_3_CN. After the reaction for 5 min, the decomposition of the G*^i^*^Bu·dibe^ derivative **10** was observed by TLC analysis. The decomposed compounds did not involve 3´,5´-*O*-bis(*tert*-butyldimethylsilyl)-2´-deoxyguanosine. The side product (peak b) might be an incompletely deprotected dG derivative or a compound having a moiety derived from the *i*Bu·dibe group.

On the other hand, cyanoethylation for the common protected monomers (C^ac^, A^pac^, and G*^i^*^Prpac^) was not observed (Figure 3ii). The results indicated that the reactivity of the *N*-monoacylated C, A, and G residues toward acrylonitrile is lower than that of the T residue. However, we found that *N*-monoacylated C and A residues were alkylated by acrylonitrile in DBU/CH_3_CN in solution phase, as shown in Table 1. The reaction of 4-*N*-acetyl-2′-deoxycytidine derivative **14** with acrylonitrile gave the 4-*N*-cyanoethylated derivative **18** in 15% yield. 6-*N*-Phenoxyacetyl-2′-deoxyadenosine derivative **15** was slightly alkylated under the same conditions to give the 6-*N*-cyanoethylated derivative **19** in 2% yield. Therefore, there is a possibility that the common protected monomers C^ac^ and A^pac^ are alkylated by acrylonitrile on the ODN synthesis even if the C and A residues are protected by the monoacylated protecting group when DBU was used. It seems that the fully protected approach is important for the C and A residues to avoid the cyanoethylation. Cyanoethylation of a 2-*N*-phenoxyacetyl-2′-deoxyguanosine derivative was not detected by TLC and mass analysis after 24 h. It was found that the *N*-monoacylated G residue could suppress the cyanoethylation by acrylonitrile.

In the C and A monomers, the phth group for complete protection of the amino groups was effective for avoidance of the cyanoethylation because *N*-monoacylated monomers proved to react with acrylonitrile. The *i*Bu·dibe group on the G*^i^*^Bu·dibe^ base was rapidly decomposed within 5 min during the DBU/CH_3_CN treatment required for removal of the 2-cyanoethyl group at the internucleotidic phosphate group as mentioned before but cyanoethylation for the G*^i^*^Bu·dibe^ residue was not observed under the conditions of acrylonitrile-DBU/CH_3_CN (Figure 3i). However, the fully protected species of T^Bz^, C^phth^, A^phth^, and G*^i^*^Bu·dibe^ were completely removed by treatment with concentrated NH_3_ at 55 °C for 6 h (Figure 3iii). Therefore, the full protection mode can be used when it is necessary to protect the base moieties to avoid any side reactions which are expected when the usual monoacylated base moieties are used for further modification of sugar or phosphate residues.

**Table 1:** Cyanoethylation of the common protected monomers in solution phase reaction.

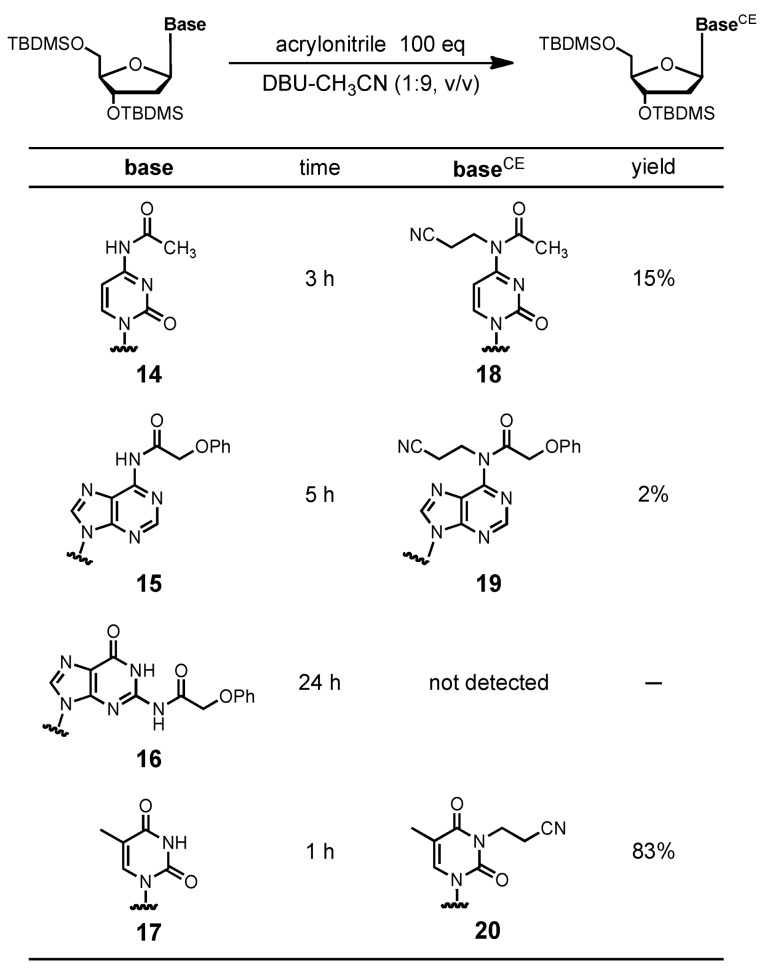

### 2.3. Synthesis of Oligonucleotides Using the Fully Protected Phosphoramidites

**Figure 4:**
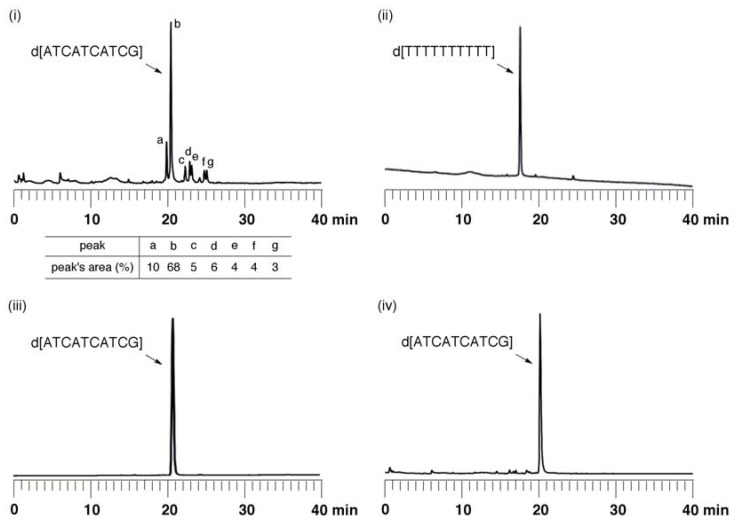
Anion-exchange HPLC profiles of ODNs obtained by using (i) the fully protected monomers (T^Bz^, C^phth^, A^phth^, and G*^i^*^Bu·dibe^); (ii) only T^Bz^ monomer and (iii) T^Bz^, C^phth^, A^phth^, and G*^i^*^Prpac^ monomers after removal of the cyaonoethyl groups of the internucleotidic phosphate groups by treatment with DBU-CH_3_CN (1:9, v/v); (iv) Anion-exchange HPLC profile of deprotection of ODN having the fully protected monomers (T^Bz^, C^phth^, A^phth^, and G*^i^*^Bu·dibe^) by concentrated NH_3_.

To evaluate if the full protection of the base residues was effective for the oligonucleotide synthesis, we synthesized an ODN with the fully protected monomers using crosslinked polystyrene beads (UnyLinker™ NittoPhase^®^). Selective removal of the 2-cyanoethyl groups at the internucleotidic phoshate groups was performed by treatment with DBU/CH_3_CN (1:9, v/v) for 1 min [35,36]. The successive release of the base-protected oligomer from the resin and complete deprotection of the other base-labile protecting groups were simultaneously conducted by treatment with concentrated ammonia at 55 °C for 14 h. The mixture thus obtained was analyzed by anion-exchange HPLC (Figure 4i). The result showed that the desired ODN appeared as the main product. In contrast, several minor products were also observed in this HPLC analysis. It was expected that treatment of dG*^i^*^Bu·dibe^ with DBU/CH_3_CN produced these byproducts, as indicated the previous section. To confirm this expectation, we prepared base-protected T_10_ and ATCATCATCG derivatives, which were synthesized on the same resins by use of T^Bz^, C^phth^, A^phth^, and G*^i^*^Prpac^ phosphoramidite building blocks. In the latter oligomer, the G*^i^*^Prpac^ monomer unit was used in place of G*^i^*^Bu·dibe^. These protected ODNs were treated with DBU-CH_3_CN under the same conditions followed by treatment with concentrated NH_3_. The analysis by HPLC suggested that the corresponding ODNs were obtained as single products and the minor products were not produced as observed in the reaction using ODN containing the G*^i^*^Bu·dibe^ monomer (Figures 4ii, iii). When a base-protected ATCATCATCG oligomer on the resin having fully protected monomers (T^Bz^, C^phth^, A^phth^, and G*^i^*^Bu·dibe^) was deprotected by treatment with concentrated NH_3_ at 55 °C for 6 h, the desired ODN was obtained without side products (Figure 4iv).

**Figure 5:**
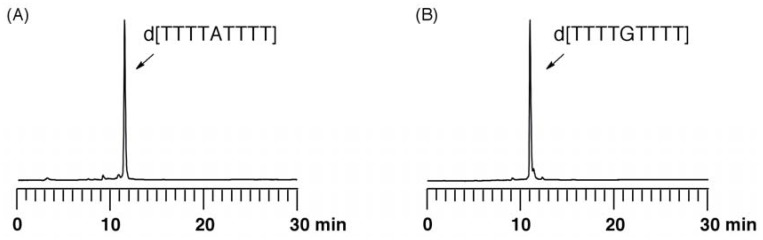
Analysis of depurination using ODNs containing (A) A^phth^ or (B) G*^i^*^Bu·dibe^ by reverse-phase HPLC.

When a longer ODN is synthesized in the solid phase, there are considerable concerns in addition to cyanoethylation at the nucleobases. One expected problem is the depurination of the A and G residues under acidic conditions, which is required for the repeated removal of the DMTr group during chain elongation. In the synthesis of a longer ODN, the total time of the repeated acidic treatments increases with chain length. Therefore, we examined the stability of the glycosyl bonds of the fully protected A^phth^ and G*^i^*^Bu·dibe^ bases in 3% TCA/CH_2_Cl_2_, which is used for removal of the DMTr group in the current DNA synthesis. To evaluate the stability of these nucleobases, we synthesized ODNs containing the fully protected monomer A^phth^ or G*^i^*^Bu·dibe^ at the central position. The synthesized ODNs on the resin were treated with 3% TCA in CH_2_Cl_2_ for 24 h. After the treatment with concentrated ammonia, the mixtures thus obtained were analyzed by reverse-phase HPLC (Figure 5). In the reaction mixture, the side products originating from the depurination of A^phth^ or G*^i^*^Bu·dibe^ were not detected. The results showed that the A^phth^ and G*^i^*^Bu·dibe^ residues were stable under acidic conditions for extended durations. These fully protecting groups on the purine bases will undoubtedly become useful for the synthesis of long chain DNAs.

### 2.4. Evaluation of Base Recognition Ability of Cyanoethylated T by T_m_ Experiments

As mentioned above, cyanoethylation of the nucleobases, especially the T residue, is well known as a side reaction in ODN synthesis. However, little attention has been devoted to study the properties of ODNs containing T^CE^. We investigated the base recognition ability of T^CE^ using *T*_m_ experiments and DNA polymerase-mediated insertion reactions.

**Scheme 4:**
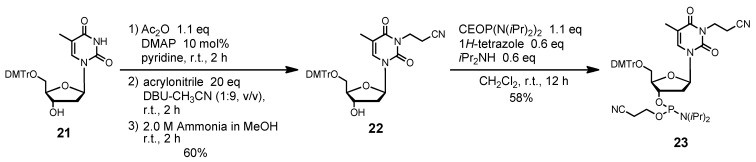
Synthesis of T^CE^ phosphoramidite unit **23**.

For the synthesis of ODN containing T^CE^, we synthesized the T^CE^ phosphoramidite unit **23** from a T derivative **21**, as shown in Scheme 4. *N*^3^-Cyanoethylation of **21** with acrylonitrile gave the cyanoethylated derivative **22** after acetylation of the 3′-hydroxy group. The phosphitylation of **22** with bis(diisopropylamino)(2-cyanoethoxy)phosphine gave the cyanoethylated phosphoramidite unit **23** in 58% yield. Subsequently, we synthesized an ODN containing T^CE^ using the phosphoramidite unit **23**, as shown in Table 2. At the deprotection step of the ODN synthesis, the *N*^3^-cyaoethyl moiety of T was found to be very stable in concentrated NH_3_.

**Table 2:** Sequences and isolated yields of ODNs containing T^CE^.

ODN	Sequence	Yield (%)	MALDI-TOF mass [M+H]^+^	
			calcd.	found
1	5′-d(CGTCTT^CE^TCTGC)-3′	48	3326.6	3329.4
2	5′-d(TTAGACT^CE^GTAACCGGTCTTCGCGCG)-3′	33	7699.3	7691.4

To clarify the base pairing properties of T^CE^ in DNA duplexes, *T*_m_ experiments were performed using duplexes formed between modified ODNs and their complementary ODNs, with matched or single-mismatched sequences at the central position, as shown in Table 3. A duplex with a matched natural T-A base pair showed a *T*_m_ value of 58.1 °C. In contrast, the *T*_m_ value of a duplex with a *N*^3^-cyanoethylated T^CE^-A base pair decreased to 36.0 °C, which is 22.1 °C lower than that of the natural T-A matched base pair. In addition, the *T*_m_ values of DNA duplexes containing the mismatched pairs (for T, C, and G) also decreased, showing a Δ*T*_m_ of −7.2 °C, −6.8 °C, and −8.4 °C, respectively. These results indicated that the base pairs having T^CE^ were unstable and the *T*_m_ values (for all bases) of DNA duplexes containing T^CE^ became similar to each other. Therefore, it was found that the sequence dependence of ODN having T^CE^ was completely lacking. A significant drop of the thermal stability of the duplex especially was observed when X was the matched A base. This is apparent because the hydrogen bonding ability of T^CE^ was lost by addition of acrylonitrile to the *N*^3^-NH group, which is an accepter site of a hydrogen bond for the A-T base pair.

**Table 3:** *T*_m_ values for DNA–DNA 11-mer duplexes containing T^CE^.

DNA–DNA duplex			d(GCA GAX AGA CG)-5′		
			5′-d(CGT CTY TCT GC)		
	Y = T		Y = T^CE^		
**X**	***T*_m_ (°C)^a^**		***T*_m_ (°C)^a^**		**Δ*T*_m_ (°C)^b^**
A	58.1		36.0		−22.1
T	43.6		36.4		−7.2
C	42.0		35.2		−6.8
G	44.0		35.6		−8.4

^a^The *T*_m_ values are accurate within ±0.5 °C. The *T*_m_ measurements were performed in a buffer containing 10 mM sodium phosphate (pH 7.0), 1 M NaCl, 0.1 mM EDTA, and 2 µM duplex. ^b^Δ*T*_m_ is the difference in the *T*_m_ values between the unmodified and modified ODNs.

### 2.5. Evaluation of the Base Recognition Ability of Cyanoethylated T by DNA Polymerase-Mediated Insertion Reactions

**Figure 6:**
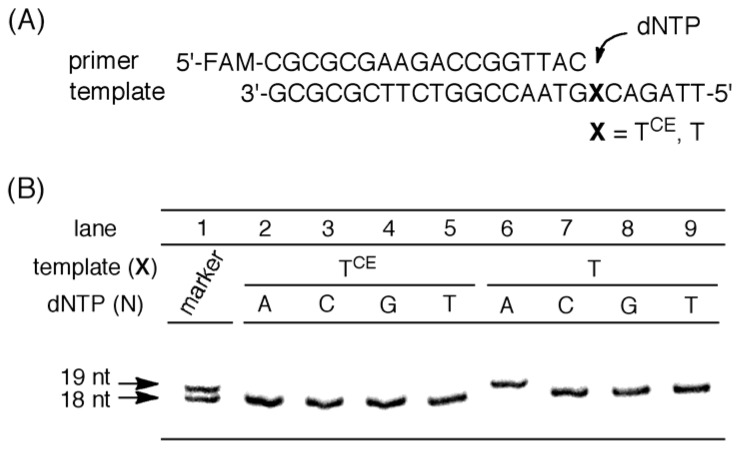
Single dNTP insertion reactions using Taq DNA polymerases. (A) Sequences of 5′-FAM labeled 18-nt primer and 25-nt templates; (B) PAGE analysis of single-insertion reactions.

To study the template specific incorporation of dNTPs using DNA polymerase, we examined the single insertion reaction of dNTPs toward the opposite T^CE^ in templates using Taq polymerase [37], a well-known thermostable enzyme used in PCR reactions [38,39]. The template incorporating T or T^CE^ at position X was annealed with a 5′-FAM-labeled 18-nt primer in the presence of DNA polymerase. Following the enzymatic reaction, the products were analyzed by PAGE, as shown in Figure 6B. Consequently, the incorporation of dATP was observed using the template containing a natural T base. In contrast, dNTPs were not incorporated into the opposite site of T^CE^ in the template by Taq DNA polymerase. It was found that *N*^3^-cyanoethylation of the T residue caused the inhibition of the base recognition by DNA polymerase and T^CE^ in the template terminated the primer extension to give no incorporation of dNTPs at the opposite site. Therefore, if synthesized ODNs including cyanoethylated species would be used as PCR templates, PCR would be stopped at the modified base to produce shorter duplicated products. Since PCR is a system that enables amplification of a trace amount of DNA, a minor contamination of *N*^3^-cyanoethylated T does not affect the whole PCR process. T^CE^, therefore, could be used as a new type of chain terminator in DNA duplications.

## 3. Experimental

### General

^1^H-, ^13^C-, and ^31^P-NMR spectra were recorded at 500, 126 and 203 MHz, respectively. The chemical shifts were measured from tetramethylsilane for ^1^H NMR spectra, CDCl_3_ (77 ppm) for ^13^C-NMR spectra and 85% phosphoric acid (0 ppm) for ^31^P NMR spectra. UV spectra were recorded on a U-2000 spectrometer. Column chromatography was performed with silica gel C-200 and a minipump for a goldfish bowl was conveniently used to attain sufficient pressure for rapid chromatographic separation. HPLC was performed using the following systems. Reversed-phase HPLC was done on a system with a 3D UV detector and a C18 column (4.6 x 150 mm). A linear gradient (0-30%) of solvent I [0.03 M ammonium acetate buffer (pH 7.0)] in solvent II (CH3CN) was used at 30 °C at a rate of 1.0 mL/min for 30 min. Anion-exchange HPLC was done on an apparatus with a 3D UV detector and a FAX column (Waters, 4.6 × 100 mm). A linear gradient (0-60%) of Solvent III [1 M NaCl and 25 mM phosphate buffer, 10% CH3CN (v/v)] in solvent IV [25 mM phosphate buffer, 10% CH3CN (v/v)] was used at 50 °C at a flow rate of 1.0 mL/min for 45 min. ESI mass was performed by use of Mariner^TM^ (PerSeptive Biosystems Inc.). MALDI-TOF mass was performed by use of Bruker Daltonics [Matrix: 3-hydroxypicolinic acid (100 mg/ml) in H_2_O-diammonium hydrogen citrate (100 mg/ml) in H_2_O (10:1, v/v)]. Compounds **1** and **21** were purchased from GeneACT, Inc. Compounds **5**, **7** [31], **9** [40], **14 [26]**,and **16 [41]** were prepared according to the published procedure. Common protected phosphoramidites were purchased from Glen Research Corporation.

*N^3^-Benzoyl-5´-O-(4, 4'-dimethoxytrityl)thymidine* (**3**). Compound **2** (2.42 g, 7.0 mmol) was rendered anhydrous by repeated coevaporation with dry pyridine (3 mL × 1) and dissolved in dry pyridine (70 mL). To the solution was added DMTrCl (3.08 g, 9.1 mmol) and the mixture was stirred at room temperature for 5 h. The reaction was quenched by addition of saturated aqueous NaHCO_3_. The mixture was partitioned between CHCl_3_ and H_2_O. The organic phase was collected, dried over Na_2_SO_4_, filtered, and evaporated under reduced pressure. The residue was chromatographed on a column of silica gel with CHCl_3_-MeOH (100:0–98:2, v/v) containing 1% Et_3_N to give the fractions containing **3**. The fractions were collected and evaporated under reduced pressure. The residue was finally evaporated by repeated coevaporation three times each with toluene and CHCl_3_ to remove the last traces of Et_3_N to give **3** (4.45 g, 98%). ^1^H-NMR (CDCl_3_) δ 1.50 (s, 3H), 2.36–2.42 (m, 2H), 3.40 (dd, 1H, *J* = 2.2 Hz, *J* = 9.5 Hz), 3.52 (dd, 1H, *J* = 2.7 Hz, *J* = 9.2 Hz), 3.81 (s, 6H), 4.05–4.06 (m, 1H), 4.60 (m, 1H), 6.39 (t, 1H, *J* = 6.6 Hz), 6.85–6.87 (m, 4H), 7.27–7.32 (m, 7H), 7.41 (d, 2H, *J* = 7.6 Hz), 7.49 (t, 2H, *J* = 7.6 Hz), 7.64 (t, 1H, *J* = 7.6 Hz), 7.70 (s, 1H), 7.94 (d, 1H, *J* = 7.6 Hz); ^13^C-NMR (CDCl_3_) δ 12.1, 41.4, 55.5, 63.7, 72.7, 85.2, 86.4, 87.3, 111.5, 113,57, 113.59, 127.5, 128.30, 128.37, 129.3, 130.3, 130.7, 131.9, 135.2, 135.50, 135.57, 135.63, 144.5, 149.5, 159.0, 163.1, 169.3. HRMS (ESI) m/z (M+Na)^+^: calcd for C_38_H_36_N_2_NaO_8_^+^ 671.2364; found, 671.2364.

*N^3^-Benzoyl-5´-O-(4,4'-dimethoxytrityl)thymidine 3'-(2-cyanoethyl N,N-diisopropylphosphor-amidite)* (**4**). Compound **3** (380 mg, 0.59 mmol) was rendered anhydrous by repeated coevaporation with dry pyridine (1 mL × 1), dry toluene (1 mL × 1), dry CH_3_CN (1 mL × 1) and dissolved in dry CH_2_Cl_2_ (6 mL). To the solution was added bis(diisopropylamino)(2-cyanoethoxy)phosphine (205 g, 0.65 mmol), 1*H*-tetrazole (25 mg, 0.35 mmol), diisopropylamine (50 μL, 0.35 mmol) and the mixture was stirred at room temperature for 3 h. The reaction was quenched by addition of saturated aqueous NaHCO_3_. The mixture was partitioned between CHCl_3_ and aqueous NaHCO_3_. The organic phase was collected, dried over Na_2_SO_4_, filtered, and evaporated under reduced pressure. The residue was chromatographed on a column of silica gel with CHCl_3_-MeOH (100:0–98:2, v/v) containing 1% Et_3_N to give the fractions containing **4**. The fractions were collected and evaporated under reduced pressure. The residue was finally evaporated by repeated coevaporation three times each with toluene and CHCl_3_ to remove the last traces of Et_3_N to give **4** (338 mg, 73%). ^1^H-NMR (CDCl_3_) δ 1.07 (d, 3H, *J* = 6.5 Hz), 1.16 (d, 9H, *J* = 5.6 Hz), 1.48 (s, 3H), 2.39 (t, 1H, *J* = 6.0 Hz), 2.48 (m, 1H), 2.55 (t, 1H, *J* = 6.0 Hz), 2.61 (m, 1H), 3.40 (t, 1H, *J* = 10.1 Hz), 3.54–3.71 (m, 5H), 3.76 (s, 6H), 4.18, 4.24 (2s, 1H), 4.75 (m, 1H), 6.42–6.45 (m, 1H), 6.86–6.89 (m, 4H), 7.24 (m, 1H), 7.32–7.37 (m, 6H), 7.43–7.48 (m, 4H), 7.59 (t, 1H, *J* = 7.3 Hz), 7.79, 7.84 (2s, 1H), 7.94 (d, 1H, *J* = 7.3 Hz); ^13^C-NMR (CDCl_3_) δ 12.1, 12.1, 20.4, 20.5, 20.6, 20.6, 24.8, 40.5, 43.4, 43.5, 43.5, 43.6, 55.5, 55.5, 55.5, 58.3, 58.4, 58.6, 63.3, 63.4, 73.7, 74.0, 85.2, 85.8, 86.1, 87.3, 111.4, 111.4, 113.5, 113.6, 117.9, 118.1, 127.5, 128.3, 128.5, 128.5, 129.4, 130.4, 130.7, 131.9, 135.3, 135.5, 135.5, 135.6, 135.6, 135.9, 144.6, 144.6, 149.6, 149.7, 159.1, 163.1, 169.5; ^31^P-NMR (CDCl_3_) δ 149.5, 149.9 (2s). HRMS (ESI) m/z (M+Na)^+^: calcd for C_47_H_53_N_4_NaO_9_P^+^ 871.3442; found, 871.3441.

*4-N-Phthaloyl-5´-O-(4,4'-dimethoxytrityl)-2´-deoxycytidine 3'-(2-cyanoethyl N,N-diisopropylphos-phoramidite)* (**6**). Compound **5** (869 mg, 1.19 mmol) was rendered anhydrous by repeated coevaporation with dry pyridine (1 mL × 1), dry toluene (1 mL × 1), dry CH_3_CN (1 mL × 1), and dissolved in dry THF (12 mL). To the solution was added phthaloyl chloride (189 μL, 1.31 mmol), diisopropylethylamine (456 μL, 2.62 mmol) at 0 °C and the mixture was stirred at room temperature for 2 h. The reaction was quenched by addition of H_2_O. The mixture was partitioned between ethyl acetate and H_2_O. The organic phase was collected, dried over Na_2_SO_4_, filtered, and evaporated under reduced pressure. The residue was chromatographed on a column of silica gel with hexane-ethyl acetate (40:60–30:70, v/v) containing 1% pyridine to give the fractions containing **6**. The fractions were collected and evaporated under reduced pressure. The residue was finally evaporated by repeated coevaporation three times each with toluene and CHCl_3_ to remove the last traces of pyridine to give **6** (846 mg, 83%). ^1^H-NMR (CDCl_3_) δ 1.04–1.17 (m, 12H), 2.40–2.45 (m, 2H), 2.62 (t, 1H, *J* = 6.3 Hz), 2.72–2.82 (m, 1H), 3.40–3.61 (m, 6H), 3.78 (s, 6H), 4.20–4.21 (m, 1H), 4.59–4.69 (m, 1H), 6.22–6.32 (m, 2H), 6.81–6.86 (m, 4H), 7.26–7.39 (m, 9H), 7.79–7.82 (m, 2H), 7.94–7.97 (m, 2H), 8.51, 8.60 (2d, 1H, *J* = 7.3 Hz); ^13^C-NMR (CDCl_3_) δ 20.1, 20.2, 20.4, 24.4, 24.5, 24.6, 40.7, 41.1, 43.1, 43.2, 43.3, 53.4, 55.2, 58.1, 58.4, 61.5, 61.9, 70.9, 71.2, 71.8, 72.1, 85.8, 86.9, 87.5, 87.6, 100.5, 113.2, 117.3, 117.5, 124.3, 127.1, 128.0, 128.1, 128.2, 130.07, 130.13, 131.4, 135.1, 135.3, 144.0, 145.2, 154.6, 158.6, 159.1, 164.9; ^31^P-NMR (CDCl_3_) δ 149.6, 150.2 (2s). HRMS (ESI) m/z (M+H)^+^: calcd for C_47_H_51_N_5_O_9_P^+^ 860.3419; found, 860.3411.

*6-N-Phthaloyl-5´-O-(4,4'-dimethoxytrityl)-2´-deoxyadenosine 3'-(2-cyanoethyl N,N-diisopropyl-phosphoramidite)* (**8**). Compound **7** (1.06 g, 1.40 mmol) was rendered anhydrous by repeated coevaporation with dry pyridine (1 mL × 1), dry toluene (1 mL × 1), dry CH_3_CN (1 mL × 1) and dissolved in dry THF (14 mL). To the solution was added phthaloyl chloride (221.7 μL, 1.54 mmol), diisopropylethylamine (1.22 mL, 7.0 mmol) at 0 °C and the mixture was stirred at room temperature for 2 h. The reaction was quenched by addition of H_2_O. The mixture was partitioned between CHCl_3_ and H_2_O. The organic phase was collected, dried over Na_2_SO_4_, filtered, and evaporated under reduced pressure. The residue was chromatographed on a column of silica gel with hexane-ethyl acetate (40:60–30:70, v/v) containing 1% pyridine to give the fractions containing **8**. The fractions were collected and evaporated under reduced pressure. The residue was finally evaporated by repeated coevaporation three times each with toluene and CHCl_3_ to remove the last traces of pyridine to give **8** (761 mg, 61%). ^1^H-NMR (CDCl_3_) δ 1.13–1.22 (m, 12H), 2.48 (t, 1H, *J* = 6.3 Hz), 2.62–2.68 (m, 3H), 2.72–2.75 (m, 1H), 3.35–3.37 (m, 1H), 3.59–3.77 (m, 9H), 4.33–4.35 (m, 1H), 4.77–4.78 (m, 1H), 6.55 (t, 1H, *J* = 5.7 Hz), 6.79–6.81 (m, 4H), 7.18–7.30 (m, 7H), 7.40 (d, 2H, *J* = 7.3 Hz), 7.82–7.83 (m, 2H), 8.01–8.02 (m, 2H), 8.34, 8.36 (2s, 1H), 8.96 (d, 1H, *J* = 3.7 Hz); ^13^C-NMR (CDCl_3_) δ 20.4, 20.6, 24.65, 24.70, 24.76, 24.8, 39.7, 39.8, 43.4, 43.5, 55.35, 55.36, 58.4, 58.5, 63.3, 63.5, 73.6, 73.7, 74.3, 74.4, 85.11, 85.13, 86.7, 113.3, 117.5, 117.6, 124.5, 127.08, 127.11, 128.0, 128.2, 128.3, 130.11, 130.15, 130.19, 130.23, 130.27, 132.1, 134.9, 135.6, 135.7, 135.8, 144.44, 144.48, 144.5, 152.5, 152.5, 153.5, 153.6, 158.7, 165.7; ^31^P-NMR (CDCl_3_) δ 149.9, 150.2 (2s). HRMS (ESI) m/z (M+H)^+^: calcd for C_48_H_51_N_7_O_8_P^+^ 884.3531; found, 884.3533.

*2-N-Isobutyryl-1,N^2^-[1,2-di(isobutyryloxy)ethylene]-3´,5´-O-bis(tert-butyldimethylsilyl)-2´-deoxy-guanosine* (**10**). 37% Glyoxal (5 mL, 40 mmol) was added to compound **9** (1.26 g, 2.0 mmol) at room temperature. The mixture was rendered anhydrous by repeated coevaporation with dry pyridine (1 mL x1) and dissolved in dry pyridine (20 mL). After the solution was stirred at room temperature for 3 h, isobutyric anhydride (6.5 mL, 40 mmol) was added, and the mixture was stirred at room temperature for 1 h. The reaction was quenched by addition of saturated aqueous NaHCO_3_. The mixture was partitioned between CHCl_3_ and H_2_O. The organic phase was collected, dried over Na_2_SO_4_, filtered, and evaporated under reduced pressure. The residue was chromatographed on a column of silica gel with hexane-ethyl acetate (90:10–70:30, v/v) to give the fractions containing **10**. The fractions were collected and evaporated under reduced pressure to give **10** (1.51 g, 98%). ^1^H-NMR (CDCl_3_) δ 0.11 (s, 12H), 0.92 (s, 18H), 1.15–1.19 (m, 12H), 1.25–1.31 (m, 6H), 2.35–2.38 (m, 2H), 2.54–2.59 (m, 2H), 3.80 (m, 2H), 4.00–4.04 (m, 2H), 4.51 (m, 1H), 6.29 (t, 1H, *J* = 6.5 Hz), 6.78 (d, 1H, *J* = 10.0 Hz), 6.83 (d, 1H, *J* = 6.3 Hz), 8.016, 8.021 (2s, 1H); ^13^C-NMR (CDCl_3_) δ -5.4, -5.3, -4.65, -4.63, 18.0, 18.46, 18.50, 18.53, 18.55, 18.57, 18.6, 18.7, 18.80, 18.83, 18.9, 19.0, 25.8, 26.1, 33.85, 33.94, 33.97, 42.6, 63.1, 72.3, 72.4, 78.6, 81.6, 84.3, 88.3, 120.6, 137.1, 148.1, 148.3, 153.7, 174.2, 174.4, 175.6. HRMS (ESI) m/z (M+H)^+^: calcd for C_36_H_62_N_5_O_9_Si_2_^+^ 764.4081; found, 764.4021.

*2-N-Isobutyryl-1,N^2^-[1,2-di(isobutyryloxy)ethylene]-2´-deoxyguanosine* (**11**). Compound **10** (1.0 g, 1.3 mmol) was dissolved in dry THF (6 mL). To the solution was added triethylamine (544 μL, 3.9 mmol) and Et_3_N·3HF (635 μL, 3.9 mmol) and the mixture was stirred at room temperature for 12 h. The mixture was partitioned between CHCl_3_-*i*PrOH (3:1, v/v) and aqueous NaHCO_3_. The organic phase was collected, dried over Na_2_SO_4_, filtered, and evaporated under reduced pressure. The residue was chromatographed on a column of silica gel with CHCl_3_-MeOH (100:0–95:5, v/v) to give the fractions containing **11**. The fractions were collected and evaporated under reduced pressure to give **11** quantitatively. ^1^H-NMR (DMSO) δ1.05–1.11 (m, 12H), 1.17–1.22 (m, 6H), 2.36–2.40 (m, 1H), 2.59–2.64 (m, 3H), 3.52–3.57 (m, 2H), 3.87 (m, 1H), 3.96 (m, 1H), 4.36 (m, 1H), 4.95–4.96 (m, 1H), 5.36 (d, 1H, *J* = 4.2 Hz), 6.23–6.24 (m, 1H), 6.70 (s, 1H), 6.77 (s, 1H), 8.29, 8.30, 8.31 (3s, 1H); ^13^C-NMR (DMSO) δ 18.7, 18.8, 18.8, 19.1, 19.1, 19.4, 19.5, 33.7, 33.8, 40.8, 61.9, 62.0, 70.9, 71.0, 78.6, 79.7, 82.2, 84.6, 84.6, 88.4, 120.4, 120.5, 139.2, 139.4, 148.2, 148.3, 148.7, 153.8, 174.8, 175.0, 175.0, 176.0. HRMS (ESI) m/z (M+H)^+^: calcd for C_24_H_34_N_5_O_9_^+^ 536.2351; found, 536.2348.

*2-N-Isobutyryl-1,N^2^-[1,2-di(isobutyryloxy)ethylene]-3´,5´-O-(4,4'-dimethoxytrityl)-2´-deoxy-guanosine* (**12**). Compound **11** (696 mg, 1.3 mmol) was rendered anhydrous by repeated coevaporation with dry pyridine (1 mL × 1), and dissolved in dry pyridine (13 mL). To the solution was added DMTrCl (533 mg, 1.6 mmol), and the mixture was stirred at room temperature for 16 h. The reaction was quenched by addition of saturated aqueous NaHCO_3_. The mixture was partitioned between CHCl_3_ and H_2_O. The organic phase was collected, dried over Na_2_SO_4_, filtered, and evaporated under reduced pressure. The residue was chromatographed on a column of silica gel with CHCl_3_-MeOH (100:0–98:2, v/v) containing 1% Et_3_N to give the fractions containing **12**. The fractions were collected and evaporated under reduced pressure. The residue was finally evaporated by repeated coevaporation three times each with toluene and CHCl_3_ to remove the last traces of Et_3_N to give **12** (657 mg, 60%). ^1^H-NMR (CDCl_3_) δ 1.14–1.19 (m, 12H), 1.26–1.31 (m, 6H), 2.55–2.59 (m, 4H), 3.80 (s, 6H), 3.92–4.03 (m, 3H), 4.74–4.75 (m, 1H), 6.28 (d, 1H, *J* = 5.4 Hz), 6.77, 6.78 (2s, 1H), 6.81–6.84 (m, 4H), 7.17 (d, 1H, *J* = 8.8 Hz), 7.22–7.32 (m, 9H), 8.60, 8.64 (2s, 1H); ^13^C -NMR (CDCl_3_) δ 18.6, 18.7, 18.9, 19.0, 19.1, 34.0, 34.1, 41.3, 41.6, 55.4, 64.2, 72.3, 78.4, 78.5, 81.8, 81.9, 84.5, 86.7, 86.8, 86.9, 113.4, 120.7, 120.8, 127.2, 128.1, 128.3, 130.2, 130.3, 130.3, 135.7, 135.8, 135.9, 137.7, 137.9, 144.7, 148.3, 148.4, 148.5, 153.9, 158.8, 174.4, 175.8, 175.9. HRMS (ESI) m/z (M+H)^+^: calcd for C_45_H_52_N_5_O_11_^+^ 838.3658; found, 838.3659.

*2-N-Isobutyryl-1,N^2^-[1,2-di(isobutyryloxy)ethylene]-3´,5´-O-(4,4'-dimethoxytrityl)-2´-deoxy-guanosine 3'-(2-cyanoethyl N,N-diisopropylphosphramidite)* (**13**). Compound **12** (210 mg, 0.25 mmol) was rendered anhydrous by repeated coevaporation with dry pyridine (1 mL × 1), dry toluene (1 mL × 1), dry CH_3_CN (1 mL × 1), and dissolved in dry CH_2_Cl_2_ (2.5 mL). To the solution was added bis(diisopropylamino)(2-cyanoethoxy)phosphine (89 μL, 0.28 mmol), 1*H*-tetrazole (11 mg, 0.15 mmol), diisopropylamine (21 μL, 0.15 mmol), and the mixture was stirred at room temperature for 14 h. The reaction was quenched by addition of saturated aqueous NaHCO_3_. The mixture was partitioned between CHCl_3_ and aqueous NaHCO_3_. The organic phase was collected, dried over Na_2_SO_4_, filtered, and evaporated under reduced pressure. The residue was chromatographed on a column of silica gel with CHCl_3_-MeOH (100:0–98:2, v/v) containing 1% Et_3_N to give the fractions containing **13**. The fractions were collected and evaporated under reduced pressure. The residue was finally evaporated by repeated coevaporation three times each with toluene and CHCl_3_ to remove the last traces of Et_3_N to give **13** (239 mg, 93%). ^1^H-NMR (CDCl_3_) δ 1.11–1.29 (m, 30H), 2.42 (m, 1H), 2.53–2.62 (m, 5H), 3.32–3.35 (m, 2H), 3.57–3.76 (m, 8H), 3.81–3.99 (m, 2H), 4.29–4.32 (m, 1H), 4.64 (m, 1H), 6.28 (m, 1H), 6.79–6.84 (m, 6H), 7.18–7.20 (m, 1H), 7.24–7.30 (m, 6H), 7.38–7.40 (m, 2H), 7.85, 7.90 (2s, 1H); ^13^C-NMR (CDCl_3_) δ 18.6, 18.7, 18.9, 19.1, 19.2, 20.4, 20.6, 24.8, 24.9, 34.0, 40.7, 40.9, 41.2, 43.5, 43.6, 55.5, 58.3, 58.4, 63.6, 63.7, 74.2, 74.3, 74.6, 78.5, 78.6, 81.7, 81.8, 84.3, 84.4, 86.0, 86.4, 86.9, 113.5, 117.6, 117.7, 120.9, 121.0, 127.2, 128.2, 128.4, 130.2, 130.3, 135.6, 135.7, 135.8, 137.2, 137.3, 144.6, 148.4, 148.5, 153.8, 158.9, 174.3, 174.4, 175.7, 175.8; ^31^P-NMR (CDCl_3_) 150.0, 150.4, 150.5 (3s). HRMS (ESI) m/z (M+H)^+^: calcd for C_54_H_69_N_7_O_12_P^+^ 1038.4736; found, 1038.4064.

*Evaluation of the resistance for addition of acrylonitrile to protected monomers.* Fully protected monomers and common protected monomers were introduced into UnyLinker™ NittoPhase^®^ by use of the ABI 392 DNA synthesizer. The protected monomer (125 nmol) loading UnyLinker™ NittoPhase^®^ were treated with acrylonitrile (0.82 μL, 12.5 μmol) in 4 mM DBU-CH_3_CN (200 μL). After the reaction for appropriate time, the reaction solution was removed by filtration. The DMTr group was removed by treatment with 3% trichloroacetic acid in CH_2_Cl_2_ (1 mL) for 1 min, and the resin was washed with CH_2_Cl_2_ (1 mL × 3), and CH_3_CN (1 mL × 3). The monomer was deprotected and released from UnyLinker™ NittoPhase^®^ by treatment with concentrated NH_3_ aq (500 µL) at 55 °C for 6 h. The polymer support was removed by filtration and washed with distilled water (1 mL × 3). The filtrate was evaporated and purified by reversed-phase HPLC.

*6-N-Phenoxyacetyl-3´,5´-O-bis(tert-butyldimethylsilyl)-2´-deoxyadenosine* (**15**). 3´,5´-*O*-Bis(*tert*-butyldimethylsilyl)-2´-deoxyadenosine (960 mg, 2.0 mmol) was rendered anhydrous by repeated coevaporation with dry pyridine (1 mL × 1) and dissolved in dry pyridine (10 mL). To the solution was added phenoxyacetic anhydride (1.37 mg, 4.8 mmol), and the mixture was stirred at room temperature for 3 h. To the reaction was added concentrated NH_3_ aq (5 mL), and the mixture was stirred at room temperature for 10 min. The mixture was partitioned between CHCl_3_ and aqueous NaHCO_3_. The organic phase was collected, dried over Na_2_SO_4_, filtered, and evaporated under reduced pressure. The residue was chromatographed on a column of silica gel with CHCl_3_-MeOH (100:0, v/v) to give the fractions containing **15**. The fractions were collected and evaporated under reduced pressure to give **15** (831 mg, 68%). ^1^H NMR (CDCl_3_) δ ^1^H NMR (CDCl_3_) δ 0.10 (s, 12H), 0.91 (s, 18H), 2.45–2.49 (m, 1H), 2.65–2.70 (m, 1H), 3.78 (dd, 1H, *J* = 2.9 Hz, *J* = 9.8 Hz), 3.87 (dd, 1H, *J* = 4.2 Hz, *J* = 9.2 Hz), 4.037–4.043 (m, 1H), 4.62–4.63 (m, 1H), 4.87 (s, 2H), 6.50 (t, 1H, *J* = 6.3 Hz), 7.08 (d, 3H, *J* = 8.3 Hz), 7.35 (t, 2H, *J* = 7.9 Hz), 8.33 (s, 1H), 8.79 (s, 1H), 9.41 (s, 1H); ^13^C-NMR (CDCl_3_) δ -5.3, -5.2, -4.6, -4.5, 18.1, 18.5, 26.0, 26.1, 40.9, 62.8, 68.6, 72.1, 84.9, 88.1, 115.0, 117.6, 122.1, 123.0, 129.8, 142.5, 148.7, 151.5, 152.3, 157.5, 167.9. HRMS (ESI) m/z (M+H)^+^: calcd for C_30_H_48_N_5_O_5_Si_2_^+^ 614.3188; found, 614.3186.

*General procedure for evaluation of cyanoethylation of compounds **14-17**.* An appropriate compound (0.30 mmol) was dissolved in DBU-CH_3_CN [3 mL, (1:9, v/v)]. To the solution was added acrylonitrile (1.96 mL, 30 mmol). After being stirred at room temperature for appropriate time, as shown in Table 1, the mixture was partitioned between CHCl_3_ and H_2_O. The organic phase was collected, dried over Na_2_SO_4_, filtered, and evaporated under reduced pressure. The residue was chromatographed on a column of silica gel with CHCl_3_-MeOH (100:0–99:1, v/v) to give the fractions containing the cyanoethylated compound. The fractions were collected and evaporated under reduced pressure to give the cyanoethylated compound. Thus, the following compounds **18**–**20** were obtained.

*4-N-Acetyl-4-N-2-cyanoethyl-3´,5´-O-bis(tert-butyldimethylsilyl)-2´-deoxycytidine* (**18**). The compound **18** was obtained (25.3 mg, 15%).^ 1^H NMR (CDCl_3_) δ 0.11 (s, 12H), 0.90 (s, 18H), 2.13–2.18 (m, 1H), 2.47 (s, 3H), 2.52–2.57 (m, 1H), 2.84–2.93 (m, 2H), 3.78 (dd, 1H, *J* = 2.6 Hz, *J* = 10.8 Hz), 3.95–3.97 (m, 2H), 4.26 (t, 2H, *J* = 6.6 Hz), 4.38–4.41 (m, 1H), 6.61–6.23 (m, 1H), 6.88 (d, 1H, *J* = 7.3 Hz), 8.39 (d, 1H, *J* = 7.6 Hz);^ 13^C-NMR (CDCl_3_) δ -5.4, -5.3, -4.8, -4.4, 17.5, 18.1, 18.5, 22.6, 25.8, 26.0, 42.3, 42.4, 61.8, 70.0, 87.1, 88.0, 99.3, 144.0, 154.9, 164.2, 172.1. HRMS (ESI) m/z (M+H)^+^: calcd for C_26_H_47_N_4_O_5_Si_2_^+^ 551.3080; found, 551.3079.

*6-N-2-Cyanoethyl-6-N-phenoxyacetyl-3´,5´-O-bis(tert-butyldimethylsilyl)-2´-deoxyadenosine* (**19**). The compound **19** was obtained (3.4 mg, 2%).^ 1^H NMR (CDCl_3_) δ 0.12 (s, 12H), 0.92 (s, 18H), 2.46–2.51 (m, 1H), 2.57–2.62 (m, 1H), 2.93 (t, 2H, *J* = 7.3 Hz), 3.79 (dd, 1H, *J* = 2.7 Hz, *J* = 9.9 Hz), 3.89 (dd, 1H, *J* = 3.9 Hz, *J* = 9.3 Hz), 4.05–4.06 (m, 1H), 4.57–4.60 (m, 2H), 4.62–4.64 (m, 1H), 5.17 (d, 2H, *J* = 3.9 Hz), 6.52 (t, 1H, *J* = 6.2 Hz), 6.67 (d, 2H, *J* = 8.1 Hz), 6.93 (t, 1H, *J* = 7.3 Hz), 7.20 (t, 2H, *J* = 7.9 Hz), 8.42 (s, 1H), 8.70 (s, 1H); ^13^C-NMR (CDCl_3_) δ -5.3, -5.2, -4.6, -4.5, 17.3, 18.2, 18.6, 25.9, 26.1, 41.7, 43.6, 62.8, 68.9, 71.9, 84.9, 88.3, 114.6, 117.6, 121.7, 125.5, 129.6, 142.7, 151.4, 151.6, 152.7, 157.7, 170.8. HRMS (ESI) m/z (M+H)^+^: calcd for C_33_H_51_N_6_O_5_Si_2_^+^ 667.3454; found, 667.3454.

*N^3^-(2-Cyanoethyl)-3´,5´-O-bis(tert-butyldimethylsilyl)thymidine* (**20**). The compound **20** was obtained (127.3 mg, 83%). ^1^H NMR (CDCl_3_) δ 0.11 (s, 12H), 0.90 (s, 18H), 1.93 (s, 3H), 1.95–2.03 (m, 1H), 2.25–2.28 (m, 1H), 2.73–2.76 (m, 2H), 3.76 (d, 1H, *J* = 11.0 Hz), 3.86 (d, 1H, *J* = 11.5 Hz), 3.95 (m, 1H), 4.24–4.29 (m, 2H), 4.39 (m, 1H), 6.34 (t, 1H, *J* = 6.8 Hz), 7.50 (s, 1H); ^13^C-NMR (CDCl_3_) δ -5.3, -5.2, -4.7, -4.5, 13.3, 16.2, 18.1, 18.5, 25.9, 26.1, 36.8, 41.6, 63.1, 72.4, 85.8, 88.1, 110.2, 117.3, 134.3, 150.6, 163.1. HRMS (ESI) m/z (M+H)^+^: calcd for C_25_H_46_N_3_O_5_Si_2_^+^ 524.2971; found, 524.2913.

*Synthesis of ODN using fully protected monomers:* The synthesis of ODN d[ATCATCATCG] by use of an ABI 392 DNA synthesizer was carried out. After chain elongation was finished, the fully protected ODN (125 nmol) on UnyLinker™ NittoPhase^®^ beads was treated with DBU-CH_3_CN [200 mL, (1:9, v/v)] for 1 min to remove the 2-cyanoethyl groups from the phosphate moieties. The ODN was deprotected and released from the resin by treatment with concentrated NH_3_ aq (500 µL) at 55 °C for 14 h. The polymer support was removed by filtration and washed with distilled water (1 mL × 3). The filtrate was evaporated and purified by anion-exchange HPLC. MALDI-TOF Mass (M+H)^+^: calcd for C_97_H_125_N_35_O_58_P_9_^+^ 2986.55; found 2987.91.

*Evaluation of depurination using ODNs containing A^phth^ or G^iBu·dibe^*. ODN d[TTTT**X**TTTT] (**X** = A^phth^ or G^iBu·dibe^) (0.5 µmol) on UnyLinker™ NittoPhase^®^ beads was treated with 3% trichloroacetic acid in CH_2_Cl_2_ (1 mL) for 24 h. After the filtration of the reaction solution, the resin was washed with CH_2_Cl_2_ (1 mL × 3) and CH_3_CN (1 mL × 3). The ODN was deprotected and released from UnyLinker™ NittoPhase^®^ by treatment with concentrated NH_3_ aq (500 µL) at 55 °C for 6 h. The polymer support was removed by filtration and washed with distilled water (1 mL × 3). The filtrate was evaporated and analyzed by reversed-phase HPLC. d[TTTTA^phth^TTTT]: MALDI-TOF Mass (M+H)^+^ calcd for C_90_H_118_N_21_O_59_P_8_^+^ 2684.48; found 2686.58. d[TTTTG*^i^*^Bu·dibe^TTTT]: MALDI-TOF Mass (M+H)^+^ calcd for C_90_H_118_N_21_O_60_P_8_^+^ 2700.47; found 2702.94.

*N^3^-(2-Cyanoethyl)-5´-O-(4, 4'-dimethoxytrityl)thymidine* (**22**). Compound **21** (437 mg, 0.80 mmol) was rendered anhydrous by repeated coevaporation with dry pyridine (1 mL × 1), and dissolved in dry pyridine (8 mL). To the solution was added acetic anhydride (83 μL, 0.88 mmol), *N*,*N*-dimethtylaminopyridine (10 mg, 0.08 mmol), and the mixture was stirred at room temperature for 2 h. The reaction was quenched by addition of saturated aqueous NaHCO_3_. The mixture was partitioned between CHCl_3_ and aqueous NaHCO_3_. The organic phase was collected, dried over Na_2_SO_4_, filtered, and evaporated under reduced pressure. The residue was dissolved in DBU-CH_3_CN (8 mL, 1:9, v/v), and to the solution was added acrylonitrile (1.05 mL, 16.0 mmol). After being stirred at room temperature for 2 h, the mixture was partitioned between CHCl_3_ and H_2_O. The organic phase was collected, dried over Na_2_SO_4_, filtered, and evaporated under reduced pressure. To the residue was added 2.0 M ammonium in MeOH (10 mL), and the mixture was stirred at room temperature for 2 h. The mixture was evaporated under reduced pressure, chromatographed on a column of silica gel with CHCl_3_-MeOH (100:0–98:2, v/v) containing 1% Et_3_N to give the fractions containing **22**. The fractions were collected and evaporated under reduced pressure. The residue was finally evaporated by repeated coevaporation three times each with toluene and CHCl_3_ to remove the last traces of Et_3_N to give **22** (289 mg, 60%). ^1^H NMR (CDCl_3_) δ 1.50 (s, 3H), 2.34–2.37 (m, 1H), 2.41–2.42 (m, 1H), 2.76 (t, 2H, *J* = 7.1 Hz), 3.39 (dd, 1H, *J* = 2.9 Hz, *J* = 10.3 Hz), 3.50 (dd, 1H, *J* = 3.2 Hz, *J* = 8.9 Hz), 3.80 (s, 6H), 4.05–4.06 (m, 1H), 4.26–4.30 (m, 2H), 4.59 (m, 1H), 6.42 (t, 1H, *J* = 6.7 Hz), 6.85 (d, 4H, *J* = 8.5 Hz), 7.25–7.32 (m, 7H), 7.40 (d, 2H, *J* = 7.3 Hz), 7.61 (s, 1H); ^13^C-NMR (CDCl_3_) δ 12.6, 16.2, 36.8, 41.2, 55.4, 63.5, 72.5, 85.5, 86.2, 87.2, 110.6, 113.5, 117.4, 127.3, 128.2, 128.3, 129.2, 130.2, 134.4, 135.41, 135.47, 144.4, 150.7, 158.9, 163.1. HRMS (ESI) m/z (M+Na)^+^: calcd for C_34_H_35_N_3_NaO_7_^+^ 620.2367; found, 620.2368.

*N^3^-(2-Cyanoethyl)-5´-O-(4, 4'-dimethoxytrityl)thymidine* *3'-(2-cyanoethyl N, N-diisopropylphosphramidite)* (**23**). Compound **22** (239 mg, 0.40 mmol) was rendered anhydrous by repeated coevaporation with dry pyridine (1 mL × 1), dry toluene (1 mL × 1), dry CH_3_CN (1 mL × 1), and dissolved in dry CH_2_Cl_2_ (4 mL). To the solution was added bis(diisopropylamino) (2-cyanoethoxy)phosphine (123 μL, 0.44 mmol), 1*H*-tetrazole (17 mg, 0.24 mmol), diisopropylamine (34 μL, 0.24 mmol), and the mixture was stirred at room temperature for 12 h. The reaction was quenched by addition of saturated aqueous NaHCO_3_. The mixture was partitioned between CHCl_3_ and aqueous NaHCO_3_. The organic phase was collected, dried over Na_2_SO_4_, filtered, and evaporated under reduced pressure. The residue was chromatographed on a column of silica gel with hexane-ethyl acetate (70:30–50:50, v/v) containing 1% Et_3_N to give the fractions containing **23**. The fractions were collected and evaporated under reduced pressure. The residue was finally evaporated by repeated coevaporation three times each with toluene and CHCl_3_ to remove the last traces of Et_3_N to give **23** (179 mg, 58%). ^1^H NMR (CDCl_3_) δ 1.07 (d, 3H, *J* = 6.8 Hz), 1.17–1.19 (m, 9H), 1.44 (s, 3H), 2.35–2.37 (m, 1H), 2.43 (t, 1H, *J* = 6.2 Hz), 2.49–2.58 (m, 1H), 2.63 (t, 1H, *J* = 6.2 Hz), 2.76 (t, 2H, *J* = 7.1 Hz), 3.32–3.35 (m, 1H), 3.48–3.68 (m, 5H), 3.80, 3.81 (2s, 6H), 4.15–4.19 (m, 1H), 4.26–4.28 (m, 2H), 4.66–4.68 (m, 1H), 6.40–6.44 (m, 1H), 6.83–6.86 (m, 4H), 7.25–7.31 (m, 7H), 7.40-7.42 (m, 2H), 7.65, 7.69 (2s, 1H); ^13^C-NMR (CDCl_3_) δ 12.5, 16.2, 20.3, 20.4, 20.55, 20.61, 24.60, 24.66, 24.69, 24.7, 24.8, 36.8, 43.35, 43.43, 43.5, 55.41, 55.43, 58.2, 58.4, 63.1, 63.3, 73.5, 73.6, 73.8, 74.0, 85.6, 85.7, 85.9, 87.1, 110.6, 113.4, 117.4, 117.5, 117.7, 127.32, 127.35, 128.1, 128.3, 128.4, 130.29, 130.31, 130.3, 134.47, 134.51, 135.46, 135.51, 144.4, 150.67, 150.70, 158.9, 163.1; ^31^P NMR (CDCl_3_) 149.7, 150.1 (2s). HRMS (ESI) m/z (M+Na)^+^: calcd for C_42_H_50_N_5_NaO_8_P^+^ 820.3446; found, 820.3450.

*Enzymes and ODNs*. AmpliTaq Gold DNA polymerase was purchased from applied biosystems^TM^, and dNTPs were purchased from Takara Bio, Inc. ODNs used in enzyme reactions and *T*_m_ experiments were purchased from Sigma-Aldrich Japan. ODNs 1 and 2 in Table 2 were synthesized by use of an ABI 392 DNA synthesizer.

*T_m_ experiments*. An appropriate ODN (2 μM) and its complementary 2 μM ssDNA 11-mer were dissolved in a buffer consisting of 1 M NaCl, 10 mM sodium phosphate, and 0.1 mM EDTA adjusted to pH 7.0. The solution was maintained at 80 °C for 10 min for complete dissociation of the duplex to single strands and cooled at the rate of UV-1700^TM^ (Shimadzu) by increasing the temperature at the rate of 0.5 °C/min. During this process of annealing and melting, the absorption at 260 nm was recorded and used to draw UV melting curves. The *T*_m_ value was calculated as the temperature at which the first derivative of the UV melting curve had a maximum.

*Single dNTP Insertion Reaction Using Taq DNA Polymerase.* A mixture containing PCR Gold buffer, 1.8 mM MgCl_2_, and 0.25 unit AmpliTaq Gold DNA polymerase was incubated at 95 °C for 3 min and slowly cooled to room temperature. To the mixture was added 100 nM (final concentration) 5′-FAM-labeled primer/template, and 10 μM (final concentration) dNTP (N = A, C, G, or T). The mixture (10 μL) was incubated at 74 °C for 10 min, and the reactions were terminated by adding 30 μL of stop solution (95% formamide, 20 mM EDTA). After being gently vortexed, the samples were separated by electrophoresis using 20% denaturing polyacrylamide gel containing 7 M urea and visualized by Fujifilm FLA-7000.

## 4. Conclusions

In this study, we have synthesized the fully protected monomers (T^Bz^, dC^phth^, dA^phth^, and dG*^i^*^Bu·dibe^), and an ODN using these monomers. The T^Bz^ monomer can be obtained efficiently from T in three steps. The dC^phth^ and dA^phth^ monomers were obtained from the corresponding *N*-unprotected phosphoroamidite units by facile acylation. The full protection of dG was achieved and used in an addition reaction with glyoxal at the *N*^1^- and 2-*N*-amino groups. To examine the stability of the fully protected monomers toward addition of acrylonitirile, we carried out the reaction of these monomers with excess acrylonitirile in DBU/CH_3_CN. As a result, T^Bz^ suppressed the addition of acrylonitrile. Full deprotection of the protecting groups used in the oligonucleotide synthesis revealed the presence of side reactions associated with dG*^i^*^Bu·dibe^. Further improvement was necessary to avoid such side reactions. In contrast, it was found that an ODN having T^CE^ formed an unstable DNA duplex in comparison with the unmodified DNA duplex. In addition, T^CE^ on the template exhibited complete inhibitory effect on chain elongation of a primer in DNA polymerase reactions.
